# Cultural Adaptation and Validation of the Premature Infant Pain Profile-Revised (PIPP-R) Pain Measurement Scale: Research Protocol

**DOI:** 10.3390/ijerph191912338

**Published:** 2022-09-28

**Authors:** Irene Núñez-López, Laura Collados-Gómez, Raquel Abalo, Patricia Martínez-Pérez, Álvaro Moreno-Vicente, María-Gema Cid-Expósito

**Affiliations:** 1 Neonatal Intensive Care Department, Hospital Universitario 12 de Octubre, Madrid (H12O), 28041 Madrid, Spain; 2 Doctoral Program in Health Sciences, International PhD School, Rey Juan Carlos University (URJC), 28008 Madrid, Spain; 3 Department of Nutrition, Faculty of Biomedicine and Nursing, Universidad Europea de Madrid, 28670 Madrid, Spain; 4 Invecuid Care Research Group, Instituto de Investigación Sanitaria Hospital 12 de Octubre (imas12), 28041 Madrid, Spain; 5 Department of Basic Health Sciences, Faculty of Health Sciences, Rey Juan Carlos University (URJC), 28922 Alcorcón, Spain; 6 High Performance Research Group on Pathophysiology and Pharmacology of the Digestive Tract (NeuGut-URJC), Rey Juan Carlos University (URJC), 28922 Alcorcón, Spain; 7 R & D & I Unit Associated with the Institute of Medicinal Chemistry (IQM), Spanish National Research Council (CSIC), 28006 Madrid, Spain; 8 Spanish Pain Society Working Group on Basic Sciences in Pain and Analgesia, 28046 Madrid, Spain; 9 Spanish Pain Society Working Group on Cannabinoids, 28046 Madrid, Spain; 10 Department of Nursing, School of Health Sciences, Rey Juan Carlos University (URJC), 28922 Madrid, Spain

**Keywords:** pain, neonate, validation, PIPP-R

## Abstract

Introduction: The main objective of this study is to validate the PIPP-R scale (Premature Infant Pain Profile-Revised) for measuring neonatal pain in the Spanish hospital setting. Materials and Methods: The original scale will be translated from English into Spanish and a consensus translation will be prepared by the research team, which will be back-translated from Spanish into English. The content validity of the Spanish version of the scale will be measured using the Delphi method. Subsequently, a multicenter observational study will be conducted to assess construct validity, internal consistency, and intra-observer and inter-observer agreement. Pain will be assessed by comparing scores for a specific non-painful procedure with those for a specific painful procedure. The sample will include 300 subjects in intensive care and intermediate care units, who will be equally distributed among the participating hospitals. The subjects will be stratified into three groups by gestational age. Discussion: The original version of the PIPP-R scale is useful for objectively assessing neonatal acute and procedural pain from a gestational age of 25 weeks and over. It is important to culturally adapt the original validated scale and to test its validity and reliability in the Spanish healthcare context. The results of this study may represent significant progress in pain management.

## 1. Introduction

In the neonatal stage, from birth to 28 days of life, pain is a complex biological, psychological, and social phenomenon. Valid, reliable assessment instruments are thus necessary given the multidimensional nature of pain, its individual, subjective nature, and the inability of neonates to verbally express the intensity of their pain.

It has been established that neonatal patients are capable of perceiving pain in the same way as adults [1]. However, their inability to verbalize pain makes it impossible to assess and manage it. If pain cannot be quantified, it remains unaddressed, and the patient will continue to experience it.

Pain assessment is the cornerstone of effective pain management. The American Academy of Pediatrics (AAP) position statement on neonatal pain [2] stipulates that pain prevention in newborns (NBs) should be a goal of all healthcare professionals involved with these patients. All facilities caring for NBs should implement a pain prevention program that includes strategies to minimize the number of painful procedures performed and a pain assessment and management plan that includes routine pain assessment. The APP suggests the use of at least one of the five validated scales, including the PIPP-R scale.

However, this is currently not uniformly implemented. A Europe-wide study led by the EUROPAIN Survey Working Group [3] concludes that measurement of pain in 243 neonatal intensive care units (NICUs) in 18 European countries stood at 58%, 45%, and 30% in the respective study groups. These data are considered worrying, as pain assessment should be the standard of care for all admitted NBs. Surprisingly, units that use sedation and analgesia on a continuous basis do not perform supplementary pain assessments. The authors acknowledge that pain assessment is not an easy task and that the existence of several different scales can make it still more confusing. Meanwhile, the results of a multicenter study for Spain [4] show that only 13 NICUs had pain assessment protocols in place and the mean number of patient pain assessments per day was 2.3 (SD = 4.8). The study concludes that the majority of neonates admitted to the NICU are not assessed for pain and that many of these units do not routinely use a pain measurement scale. Among those that do, the scales used vary greatly.

There are multiple pain assessment scales for neonatal patients, which differ according to the type of pain assessed, the gestational age of the patient, and the characteristics of their items. However, currently, none of them has been validated and culturally adapted to the Spanish healthcare context. A systematic review published in 2019 [5] provides a summary of these acute pain rating scales in neonates (Table 1). The PIPP-R (Premature Infant Pain Profile- Revised) is rated as one of the scales with the lowest risk of bias.

Pain in NBs can have multiple consequences. Some are immediate, such as stress and haemodynamic instability, and others are long-term, such as allodynia, peripheral sensitisation, reactivity to pain continuing after discharge, altered somatisation, and changes in the subcortical white matter of the brain [6,7,8,9].

A study conducted in France in 2005 and 2006 (EPIPPAIN) showed that painful procedures are very frequent in hospitalized NBs. For the 430 NBs included in the study, there was a mean of 17 painful procedures per day per sick NB, most of which were not accompanied by analgesia [10]. Similar results were reported in a systematic review [11] of 18 studies, in which 7.5–17.3 painful procedures were identified each day for every hospitalized NB.

The author of the original Premature Infant Pain Profile (PIPP) scale, Dr Bonnie Stevens, proposed the scale because of a persistent clinical problem that she had identified: inadequate measurement of neonatal pain in preterm newborns (PTNBs) [12]. She subsequently revised the scale and identified errors in pain assessment in extremely preterm newborns (EPTNB) due to the limitations of psychometric data for NBs under 32 weeks of gestation, and proceeded to validate a revised version, the PIPP-R scale [13]. The revision included changes to the physical design, more detailed instructions for use, and clarification of the scoring of gestational age (GA) and baseline behavior (BB) indicators for PTNBs and term newborns (TNB).

Neonatal patients are unable to express painful sensations verbally. As a result, they are completely dependent on the subjective judgement of their care team for the interpretation and management of their pain.

Ensuring an optimal level of comfort and reduced stress using pharmacological and non-pharmacological measures is a major challenge for neonatal unit workers. Adequate pain assessment is necessary for the correct implementation of these measures.

Pain assessments should be determined by the multidisciplinary team caring for neonates [2]. Scales based on behavioral, physiological, and contextual items help to combine the subjective impressions of several professionals with different levels of expertise into a common assessment.

The availability of a neonatal pain scale (such as the PIPP-R) that has been validated and culturally adapted to the Spanish healthcare context is crucial for the provision of high-quality care to neonatal patients at an optimal level of comfort.

The PIPP-R scale was chosen because it fits the characteristics of hospitalized neonatal patients in Spain, because its original English version has been demonstrated to be valid and reliable, and because it is the most widely used scale [5,14].

For all these reasons, and in view of the lack of a validated scale for neonatal pain assessment in hospital settings in Spain, we deem it necessary to validate the PIPP-R scale cross-culturally. This re-validation is necessary when changing the language of the original scale, as the reliability and validity of the new version must be ascertained for use in the new language population. In addition, the use of validated scales as data collection instruments in research studies improves methodological precision and facilitates comparisons with similar studies.

## 2. Materials and Methods

### 2.1. Aims

The primary objective of this study is to culturally adapt and validate the PIPP-R scale for use in the Spanish healthcare context by addressing the following research objectives:

- To culturally adapt the scale and its instructions for use in the Spanish healthcare context.
- To explore the validity of this adaptation.
- To measure its reliability.

### 2.2. Design

This is a prospective, observational, multicenter study. The recommendations of the SPIRIT Statement were followed when developing this protocol [15].

#### 2.2.1. Translation and Back-Translation

In the first phase, the aim was to produce a cultural adaptation, i.e., an equivalent translation not only of the words, but also of the concepts and technical criteria found in the questionnaire [16]. The aim was also to ensure that the construct is preserved in the translation, that it can be compared in the target culture, and that the interpretation of the measurements is adaptable and understandable in both the source and target cultures.

Consent was sought from the author of the original scale for its cross-cultural adaptation and validation into Spanish. Once permission was obtained, an independent pairwise translation was performed: the original scale and its instructions for use were translated into Spanish by a bilingual native Spanish-speaking healthcare professional and a bilingual native Spanish-speaking non-healthcare professional. Subsequently, both translators agreed on the first Spanish version. This first version was back-translated into the source language/context by a bilingual native English-speaking healthcare professional and a bilingual native English-speaking non-healthcare professional independently. A second English version was produced based on the consensus of both translators on their respective back-translations, which was then compared with the original English scale by the research team for similarity/equivalence, producing the final English version of the back-translated scale.

#### 2.2.2. Content Validity

In the second phase, to ensure that the items represent the phenomenon to be measured, content validation was carried out through evaluation by a group of experts.

A heterogeneous group of 10 experts in the fields of pain and neonatal care, with different professions, levels of training and experience, and from a variety of locations was selected according to adapted translation of Quatrini’s criteria for expertise based on the literature [17] (Table 2). Only experts who scored above 5 were included.

The characteristics relating to the relevance, ambiguity, clarity, and simplicity of the items on the scale were rated from 1 to 4 using an ad-hoc online survey on the LimeSurvey^®^ platform. This platform was also used to assess the experts’ suggestions via the Delphi method, giving them the opportunity to leave their opinions/suggestions for each item for potential modification.

The Content Validity Index (CVI) was used to calculate this in accordance with Lynn and Yahgmale [18,19].

To assess the results, the CVI of each item (I-CVI) and the CVI of the scale as a whole (S-CVI) were estimated as per Polit and Beck [20]. The I-CVI was calculated by dividing the number of experts who awarded the item a score of 3–4 by the total number of experts; and the S-CVI was calculated by averaging the I-CVIs of all items in the scale.

In keeping with the Delphi method, the initial survey, containing closed and open questions, was sent to the experts. An initial analysis of their answers was performed by the research team and items were modified as appropriate. Subsequently, the questionnaire was sent out for a second time with the modifications and the results of the first assessment, highlighting the conclusions of all experts. After their responses were received, a further analysis was carried out and a final report was produced and sent to all participating experts.

#### 2.2.3. Multicenter Study

In the third phase, a prospective, observational, multicenter study will be conducted in hospitals in the Spanish National Health System. These will be level II and III hospitals according to the classification of the Spanish Society of Neonatology (SENeo). The study will begin in September 2022 and will end when the sample is complete.

At the same time, the scale will be assessed for feasibility with the nurses collaborating in the multicenter study.

#### 2.2.4. Study Setting

The study setting will be the neonatal units at the Spanish Health System hospitals classified as level II and III according to the SENeo [21], whose patients are PTNBs and TNBs. 

These hospitalization units are divided into Intensive Care and Intermediate or Basic Care, defined in the Spanish Royal Decree 1277/2003 of 10 October as follows:

- Intermediate or Basic Neonatal Care: care of NBs of gestational age above 32 weeks or weighing more than 1500 grams with a mild condition requiring special intermediate care techniques.
- Neonatal Intensive Care: care of NBs with a life-threatening medical/surgical condition requiring special treatment and care on a continuous basis.

The following hospitals have so far agreed to participate: 12 de Octubre University Hospital in Madrid, University Hospital Complex in A Coruña, Donostia University Hospital, La Fe University and Polytechnic Hospital in Valencia, Clinical University Hospital in Valencia, Santa Lucía General University Hospital in Cartagena, Río Ortega University Hospital in Valladolid, and Lozano Blesa Clinical Hospital in Zaragoza. We are still awaiting confirmation from other hospitals.

#### 2.2.5. Participants

The study participants will be neonatal patients hospitalized in several of the aforementioned units from the start of the study until the sample is complete.

The inclusion criteria for participants will be: (a) PTNBs older than 25 weeks gestational age at birth; (b) receiving a painful procedure for diagnostic, therapeutic, or care purposes; (c) receiving a non-painful procedure within 24 h of the painful procedure; (d) parental consent; (e) haemodynamically stable neonates. Patients receiving analgesia can participate, but this will be taken into consideration at a later stage.

Patients will be recruited as they meet the inclusion criteria and once a member of the research team has informed their parents about the study, given them the information sheet and informed consent form, and one of the parents has returned the informed consent form with their signature.

The exclusion criterion will be (a) patients being treated with muscle relaxants that may interfere with pain responses.

#### 2.2.6. Sample

A variety of methods are used to calculate the necessary sample size in scale validation studies, and many of the studies consulted do not report on the criteria used to do so. The samples in these studies range from 30 to 100 subjects.

To calculate the sample size, Streiner [22] suggests using at least 10 participants per scale item to be validated, i.e., 70 participants in the case of this study.

After discussion, the research team decided to select 300 subjects divided into 100 subjects per group, as recommended by several authors [23], who propose this number of subjects to make it possible to perform the factor analysis necessary to assess the construct validity of the scale. This is intended to provide greater patient heterogeneity.

The number of subjects from each participating hospital will be calculated based on the number of admissions in the preceding year.

The neonates included in the study will be divided into three homogeneous groups for analysis based on their corrected gestational age, which is to be calculated in weeks and days of postmenstrual gestational age plus weeks and days of life.

- Very and extremely premature infant: 25–31 + 6 weeks gestational age.
- Moderately premature infant: 32–36 + 6 weeks gestational age.
- Term infant: >37 weeks gestational age.

#### 2.2.7. Study Measures

##### Outcome Variables: Pain Score as Measured by the PIPP-R scale in Spanish

The PIPP-R scale for the assessment of neonatal acute and procedural pain [13] consists of 7 multidimensional items (Table S1) and a table detailing instructions for use (Table S2). Three of the scale items are behavioral (facial gestures), two are physiological (heart rate and oxygen saturation), and two are contextual (corrected gestational age and baseline behavior). Behavioral and physiological items are scored numerically on a four-point scale (ranging from 0 to 3) to reflect changes in each variable from reference or baseline values. Contextual items are also scored on a four-point scale (ranging from 0 to 3) before the onset of pain (before manipulation) but will only be taken into consideration if the sum of the five aforementioned items is greater than 0. This will ensure that an EPTNB sleeping peacefully is not scored 6 points and no artificially higher scores will be obtained for pain assessment before pain occurs. A score between 0 and 6 will be considered as no pain or mild pain; a score between 7 and 12 will be considered as moderate pain; and a score between 13 and 21 will be considered as severe pain [12].

#### 2.2.8. Validation of the Scale

Construct validity: difference between the total pain scores for the painful procedure and for the non-painful procedure as measured using the Spanish version of the PIPP-R.

Inter-observer and intra-observer validity: difference between the total score given by the collaborating nurse and the total score given by a member of the research team.

##### Feasibility of the Scale: Survey of the Nurses Collaborating in the Study

Sociodemographic and clinical variables: birth weight, gestational age, sex, diagnosis on admission, admitted to the NICU or to intermediate or basic care, and days of life at the time of measurement.

Variables related to pain prevention: use of sucrose, breastfeeding or expressed breast milk, restraints, non-nutritive sucking, and/or pharmacological analgesia. All pharmacological analgesia administered two hours before the procedure shall be documented, including whether it is intermittently or continuously administered and the specific drug used. 

Variables related to painful procedures: the heel prick test, venepuncture, or dressing removal [11,24].

Variables related to non-painful procedures: Change of position or nappy change. It shall be documented whether the procedure has been carried out by the professionals in charge of the patient or by the parents.

Variables related to the nurse observer: years of experience in neonatal care, sex, age, and work unit.

All variable data will be collected in the REDCap^®^ electronic data collection notebook, which will be pilot-tested by the research team and collaborators for content validation purposes.

#### 2.2.9. Recruitment and Data Collection

**Figure 1:**
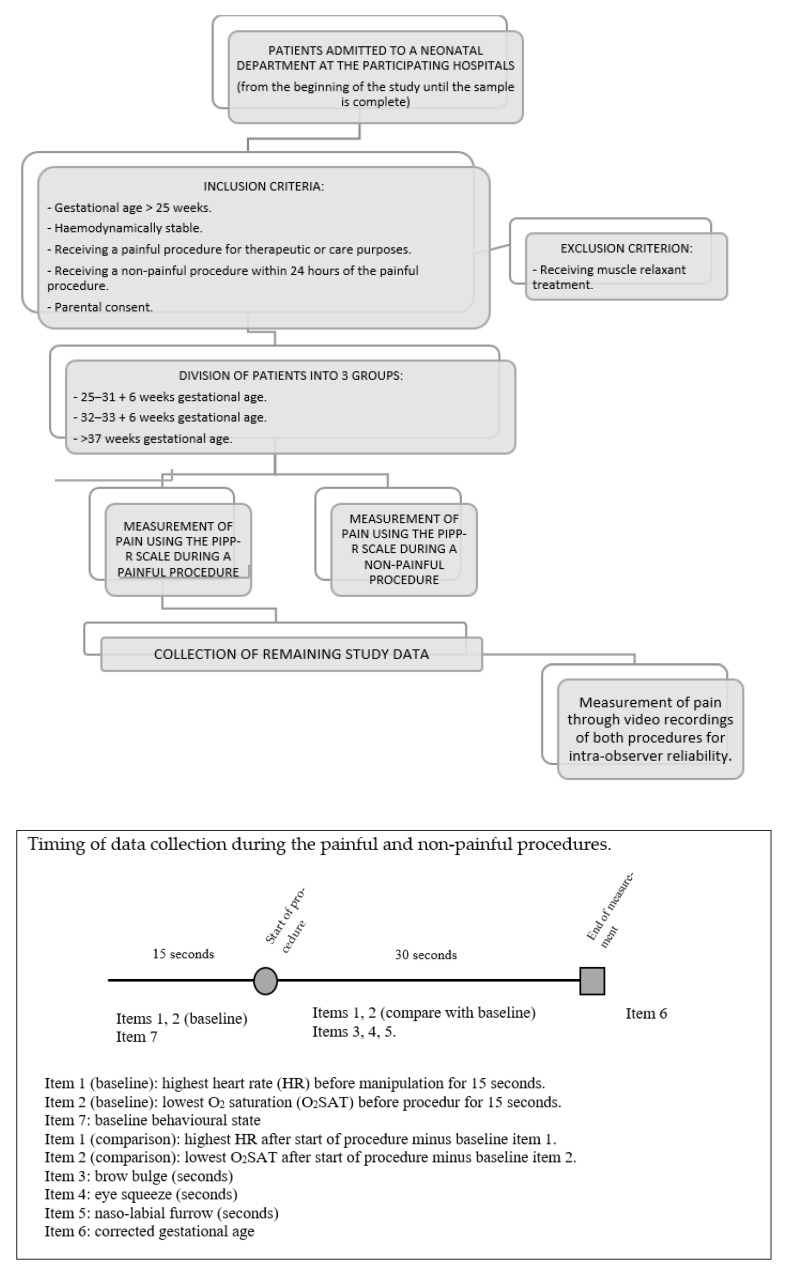
Data collection and timing diagram.

Data collection will begin once the nurses have completed their standardized training in the use of the PIPP-R scale and the data collection notebook (Figure 1). This training will preferably be face-to-face. However, if this is not possible due to the SARS-CoV-2 pandemic, online training with live workshops will be considered.

Whenever a collaborating nurse identifies a painful or non-painful procedure scheduled for a patient, she will contact her facility’s expert assessor to arrange the timing of the pain assessment and seek parental consent.

The nurse and the study facility coordinator will independently measure pain in real time at the bedside for a painful procedure and for a non-painful procedure on the same patient within 24 h of one another. Under no circumstances will a procedure be purposefully performed on a patient in order to conduct the study.

To measure pain, the patient’s heart rate and oxygen saturation must be monitored using continuous electrocardiography and pulse oximetry respectively. This will be necessary to assess the patient, as per the PIPP-R, before and during the procedure to be measured. Measuring pain with the PIPP-R scale involves the following steps:

- Observe infant for 15 s at rest (without manipulation) and assess vital sign indicators (highest heart rate and lowest oxygen saturation) and baseline behavioral state.
- Observe infant for 30 s after procedure and assess change in vital sign indicators and duration of facial actions observed.
- If the sub-total is >0, score for corrected gestational age and baseline behavioral state and calculate the total score by adding all sub-scores.

In addition, the electronic data collection notebook includes data on the collaborating nurse, the patient, and the procedure described earlier in this protocol.

Intra-observer reliability will be measured at a single facility due to the need for the necessary resources to be in place. Recording is vital, as intra-observer reliability must be measured by a single individual comparing pain assessment at the bedside with the pain assessment for the same procedure after a minimum period of two weeks. The patient’s face and vital signs will be recorded during the painful and non-painful procedures at their bedside in the neonatology department. Two Samsung Galaxy cameras will be used for this purpose, and the recordings will be transferred to a dedicated computer in the study room in the neonatology department at 12 de Octubre University Hospital in Madrid, Spain.

The patient timeline is shown in Table 3.

#### 2.2.10. Data Analysis

The descriptive analysis of quantitative variables will be carried out using means and standard deviations, as well as 95% confidence intervals, or medians and interquartile ranges if the sample is not normally distributed. Qualitative variables will be expressed as frequencies and percentages.

Construct validity, understood as the success of the instrument in representing and measuring the theoretical concept in question, will be explored using exploratory factor analysis (EFA). Values between 0.5 and 0.7 will be considered significant; values greater than 0.7 will be considered relevant [25].

Internal consistency, which measures the homogeneity of the items, will be assessed using Cronbach’s alpha coefficient. A low Cronbach’s alpha (α  <  0.70) indicates inadequate internal consistency, whereas a high Cronbach’s alpha (α  >  0.90) suggests redundancy of items [26].

Inter-observer reliability, i.e., the level of agreement between different observers of the painful procedure and the non-painful procedure, will be measured using the intraclass correlation coefficient (ICC). Values > 0.75 indicate excellent levels of reliability [25,27].

Intra-observer reliability, referring to a single observer’s level of agreement between assessments of the painful procedure and the non-painful procedure after a period of time, will also be measured using the ICC. Values > 0.75 indicate excellent levels of reliability [25,27].

The feasibility of the scale will be measured by surveying the nurses collaborating in the study using the same survey employed by the author of the original PIPP-R scale validated for the same purpose [28].

The statistical significance threshold for all analyses will be set at *p* < 0.05. Data analyses will be performed using SPSS Statistics v. 25 software (IBM, Chicago, IL, USA).

#### 2.2.11. Ethical Considerations

Prior to the study, parents and/or guardians of the neonates who are eligible to participate will be informed of the study objectives and invited to sign the informed consent form.

Confidentiality will be ensured. Participants will be assigned a unique identification code that will be used throughout the study to preserve their anonymity.

Only techniques that are needed for the neonates’ health will be performed and assessed (heel prick test, venepuncture, or other painful technique), and they will not be influenced by the conduct of this study.

The project was approved by the Ethics Committee for Research with medicinal products (ECRmp) for 12 de Octubre University Hospital (19/271) and by the Research Ethics Committee for Rey Juan Carlos University in Madrid (2406201911219).

The research team undertakes to conduct the study in accordance with current Spanish regulations, best clinical practice, and the ethical principles for medical research in humans set out in the Declaration of Helsinki.

## 3. Discussion

The validation study of the original PIPP-R scale in English [13] revealed that it is a useful tool for objectively assessing neonatal acute and procedural pain from a gestational age of 25 weeks and over. Measuring neonatal pain in other healthcare contexts with different languages and cultures is crucial. Currently, there are several culturally validated versions of this scale in other languages, including Indonesian [29], four Nordic languages [30], Brazilian Portuguese [31], and Turkish [32].

Therefore, we believe it is important to the original validated scale and to test its validity and reliability in the Spanish healthcare context. This is the rationale underpinning the study, which will be carried out using a sample of a large, heterogeneous population (from different hospitals and neonatal care units) with a range of health conditions.

The results of this study may represent significant progress in pain management [2] as well as in encouraging pain management for these patients.

Potential limitations of the study include failing to reach the estimated necessary sample size, in which case we will consider the need to include new hospitals fulfilling the study requirements.

Other limitations may be linked to training participants to use the scale, as the SARS-CoV-2 pandemic may preclude face-to-face training, or to a lack of funding, which is why intra-observer reliability will only be assessed at the patients’ primary hospitals.

## 4. Conclusions

We believe that the results of this study will prove highly relevant for the provision of comprehensive care to neonatal patients by healthcare professionals working in neonatal units, as well as for healthcare institutions.

The cross-cultural validation of a pain measurement scale for neonatal patients in the Spanish healthcare context will improve pain management in neonatal patients by optimizing the use of sedation and analgesia according to the patient’s current clinical status at all times. It will also improve the cost-effectiveness of nursing care by taking into consideration patients’ pharmacological and non-pharmacological requirements, as well as enhancing the quality of nursing care itself, as the professionals caring for these patients will possess in-depth knowledge of the tool.

Furthermore, potential sequelae could also be prevented with proper assessment and management of pain, reducing its impact on both the individual and the healthcare system as a whole.

## Figures and Tables

**Table 1:** Assessment scales for acute neonatal pain adapted from Giordano, V, 2019 [5].

Scale´s Name	Characteristics of Scale			Use to Gestational Age	
	Multidimensional	Behavioural	Number of Variable	Preterm	Term
ABC pain scale	no	yes	univariable	yes	yes
Acute Pain in Newborns	no	yes	univariable	yes	yes
Adapted COMFORT	yes	no	multivariable	yes	no
Behavioral Indicators of Infant Pain	no	yes	multivariable	yes	no
COMFORT-Behavior Scale	no	yes	multivariable	yes	yes
COVERS neonatal pain scale	yes	no	multivariable	yes	yes
CRIES Scale	yes	no	multivariable	yes	yes
Faceless Acute Neonatal Pain Scale	yes	no	multivariable	yes	no
Harrison	yes	no	multivariable	yes	yes
Infant Body Coding System	no	yes	multivariable	yes	yes
Neonatal Acute Pain Assessment Scale	yes	no	multivariable	yes	yes
Neonatal Infant Pain Scale	no	yes	multivariable	yes	yes
Neonatal Pain, Agitation and Sedation Scale	yes	no	multivariable	yes	no
Nepean Neonatal Intensive Care Unit Pain Assessment Tool	yes	no	multivariable	yes	no
Observational visual analog scale	no	yes	univariable	yes	yes
Pain assessment scale for preterm infants	yes	no	multivariable	yes	no
Premature Infant Pain Profile	yes	no	multivariable	yes	yes
Premature Infant Pain Profile Revised	yes	no	multivariable	yes	yes
Scale for Use in Newborns	yes	no	multivariable	yes	yes

**Table 2:** Expert rating base on Quatrini’s criteria [17].

Criterion	Score	Expert Rating
Doctorate	4 points	2.69
Doctoral thesis on neonatal pain	1 point	0.22
Clinical experience with neonates	1 point per year	9.22
Research projects in neonatal pain	1 point	0.67
Publications in neonatal pain	1 point	0.44
Specific training in neonatal pain	2 points	1.56
Participation in a working group on neonatal pain	1 point	0.78
Delivery of specific training in neonatal pain	2 points	0.67
Total score		

**Table 3:** Patient timeline.

	Activity/Measurement	Team Member	Estimated Completion Time	Before Assessment of Procedural Pain	During Assessment of Procedural Pain	After Assessment of Procedural Pain
Intervention	Measurement of painful procedure	Coordinator and trained professional	1 min		x	
	Measurement of non-painful procedure	Coordinator and trained professional	1 min		x	
	Pain assessment from video recordings	Coordinator and trained professional	4 min			x
Measurements	Highest baseline HR	Coordinator and trained professional	15 s		x	
	Lowest baseline O_2_SAT	Coordinator and trained professional	15 s		x	
	Baseline behaviour	Coordinator and trained professional	15 s		x	
	Highest HR during procedure	Coordinator and trained professional	30 s		x	
	Lowest O_2_SAT during procedure	Coordinator and trained professional	30 s		x	
	HR comparison (proc-baseline)	Coordinator and trained professional	10 s		x	
	O_2_SAT comparison (proc-baseline)	Coordinator and trained professional	10 s		x	
	Intensity of brow bulge (sec)	Coordinator and trained professional	30 s		x	
	Intensity of eye squeeze (sec)	Coordinator and trained professional	30 s		x	
	Intensity of naso-labial furrow (sec)	Coordinator and trained professional	30 s		x	
	Sub-total score	Coordinator and trained professional	10 s		x	
	Corrected gestational age	Coordinator and trained professional	1 min		x	
	Total score	Coordinator and trained professional	10 s		x	
Patient data	Birth weight	Coordinator	1 min	x		
	Corrected gestational age	Coordinator	1 min	x		
	Sex	Coordinator	1 min	x		
	Diagnosis on admission	Coordinator	1 min	x		
	Days of life	Coordinator	1 min	x		
Pain relief	Sucrose	Coordinator	1 min			x
	Breastfeeding	Coordinator	1 min			x
	Expressed breast milk	Coordinator	1 min			x
	Restraints	Coordinator	1 min			x
	Non-nutritive sucking	Coordinator	1 min			x
	Intermittent pharmacological analgesia 2 hours prior	Coordinator	1 min			x
	Continuous pharmacological analgesia 2 hours prior	Coordinator	1 min			x
	Drug used for pharmacological analgesia	Coordinator	1 min			x
Trained professional data	Years of experience in neonatal care	Coordinator	1 min			x
	Age	Coordinator	1 min			x
	Sex	Coordinator	1 min			x
	Hospital	Coordinator	1 min			x

## Data Availability

Data not available due to privacy and copyright protection.

## Supplementary Materials

The following supporting information can be downloaded at: https://www.mdpi.com/article/10.3390/ijerph191912338/s1, Table S1: Spanish version of the PIPP-R after assessing for content validity; Table S2: Spanish version of the PIPP-R instructions for use after assessing for content validity.
