# Supplementary material for: Cultural Adaptation and Validation of the Premature Infant Pain Profile-Revised (PIPP-R) Pain Measurement Scale: Research Protocol
Source: Int J Environ Res Public Health. 2022 Sep 28;19(19):12338. doi: 10.3390/ijerph191912338 (PMC9566023; doi:10.3390/ijerph191912338)
Supplement: Supplementary file 1 [file ijerph-19-12338-s001.zip › ijerph-1844910-supplementary.pdf]

Supplementary Table S1. Spanish version of the PIPP-R after assessing for content validity.

INDICADORES DEL NIÑO	RESULTADOS				RESULTADO DE INDICADORES DEL NIÑO
	**0**	**+1**	**+2**	**+3**	
**CAMBIOS EN FRECUENCIA CARDIACA (latidos por minuto) Valor Basal……………**	0-4	5-14	15-24	>24	
**DISMINUCIÓN EN LA SATURACIÓN DE OXÍGENO (porcentaje %) Valor Basal……….**	0-2	3-5	6-8	> 8 o incremento de FiO2	
**CEÑO FRUNCIDO (segundos)**	Ninguno (<3)	Mínimo (3-10)	Moderado (11-20)	Máximo (>20)	
**OJOS APRETADOS (segundos)**	Ninguno (<3)	Mínimo (3-10)	Moderado (11-20)	Máximo (>20)	
**SURCO NASOLABIAL MARCADO (segundos)**	Ninguno (<3)	Mínimo (3-10)	Moderado (11-20)	Máximo (>20)	
**PUNTUACIÓN SUBTOTAL**					
**EDAD GESTACIONAL CORREGIDA (semanas + días)**	≥ 36 semanas	32 semanas - 35 semanas + 6 días	28 semanas - 31 semanas + 6 días	< 28 semanas	
**COMPORTAMIENTO BASAL**	Activo y despierto	Tranquilo y despierto	Activo y dormido	Tranquilo y dormido	
**PUNTUACIÓN TOTAL**					

Supplementary Table S2. Spanish version of the PIPP-R instructions for use after assessing for content validity.

**Paso 1**	Observe al bebé durante 15 segundos en situación de reposo (sin manipulación) y evalúe los indicadores de signos vitales (valor más alto de frecuencia cardiaca, valor más bajo de saturación de oxígeno) y estado de comportamiento basal.
**Paso 2**	Observe al bebé, tras iniciar un procedimiento, durante 30 segundos y evalúe los cambios en los indicadores de signos vitales (valor más alto de frecuencia cardiaca, menor de saturación de oxígeno) y duración de los cambios en la expresión facial.<br>* Si el bebé necesita un aumento del aporte de oxígeno en cualquier momento antes o durante el proceso, recibirá una valoración de 3 en el indicador de Saturación de Oxígeno.
**Paso 3**	Calcule la puntuación subtotal. Si el resultado de la puntuación subtotal es >0, entonces puntúe la edad gestacional corregida y el comportamiento basal.<br>* Edad gestacional corregida: edad post menstrual + semanas y días de vida.<br>* Comportamiento basal: Activo (movimientos) versus Tranquilo (no movimientos)<br>Despierto (ojos abiertos) versus Dormido (ojos cerrados)
**Paso 4**	Calcule la puntuación total, sumando la puntuación subtotal + las puntuaciones de edad gestacional corregida y comportamiento basal.
